# Guideline Adherence As An Indicator of the Extent of Antithrombotic Overuse and Underuse: A Systematic Review

**DOI:** 10.5334/gh.1142

**Published:** 2022-08-12

**Authors:** Magnolia Cardona, Louise Craig, Mark Jones, Oyungerel Byambasuren, Mila Obucina, Laetitia Hattingh, Justin Clark, Paul Glasziou, Tammy Hoffmann

**Affiliations:** 1 Institute for Evidence Based Healthcare, Bond University, Gold Coast, QLD, Australia; 2 Evidence Based Practice Professorial Unit, Gold Coast University Hospital, Southport, QLD, Australia; 3 Institute of Cardiovascular and Medical Sciences, University of Glasgow College of Medical, Veterinary and Life Sciences, Glasgow, UK; 4 Gold Coast Hospital and Health Service, Southport, QLD, Australia

**Keywords:** adherence, guidelines, review

## Abstract

Thromboembolic events are a common risk in adults with atrial fibrillation, those with previous cerebrovascular accidents and undergoing emergency or elective surgeries. The widespread availability of antithrombotic agents and differing guidelines contribute to practice variations and increased risk of complications and deaths. The objective of this review was to investigate the extent of overuse and underuse of antithrombotics for primary or secondary prevention as measured by deviation from prescribing guideline recommendations. We conducted a systematic review of Medline and EMBASE for quantitative articles published between 2000 and 2021 and used a modified version of the Hoy’s risk of bias assessment tool. Here we report evidence from the past decade about wide practice variations in hospitals and primary care, and discuss clinician and patient-driven determinants of non-adherence to guidelines. Finally, we summarise implications for practice, identify enhanced ways of measuring overuse and underuse, and propose potential solutions to the measurement challenges.

## Introduction

Overdiagnosis [1] and overtreatment, low-value care [2], and unnecessary medical care [3] have been increasingly recognised in the medical literature [4] in recent years. These terms refer to the administration of a test, treatment or procedure when the consequences of doing so are not warranted, the treatment does not clearly benefit the patient [5], generates unsustainable healthcare costs, decreases healthcare quality, potentially induces demand, or deviates from and exceeds guideline recommendations without a clinically justified explanation [6]. Conversely, underuse denotes the lack of testing or provision of an effective treatment when clinically indicated [7]. Both overuse and underuse can potentially cause unintended harm.

The concepts of antithrombotic (oral and injectable anticoagulants, and antiplatelets) overuse and underuse – defined later in the methods section – have previously been debated and remain controversial [8,9] and investigated in primary studies of individual medications and specific conditions across settings [10,11]. Thromboembolic events are prevalent in adults with atrial fibrillation (AF), those with previous cerebrovascular accidents and patients undergoing emergency or elective surgeries. The wider availability of antithrombotic agents and multiple updates of guidelines contribute to practice variations that can increase complications and deaths. However, to our knowledge there are no systematic syntheses of deviation from guidelines on antithrombotic use. This may partly be due to the diversity of available medications, accepted practices across countries, and discretionary exemptions to indications. While it is acknowledged that clinical guidelines do not fit all purposes or cover all situations or patient types, they are designed to play a role in minimising practice variation [12] and supporting high standards of quality care by updating recommendations as evidence becomes available [13]. The objective of this review was to investigate the extent of contemporary overuse and underuse of antithrombotics for primary or secondary prevention as measured by deviation from prescribing guideline recommendations.

## Methods

This review follows the reporting recommendations of PRISMA 2020 [14]. As our intention was to draw a population-wide profile of the extent of the problem, our focus was groups of medications for single or multiple conditions, and single medications for groups of conditions. We chose guideline adherence to clinical guidelines as a proxy measure of overuse and underuse in this study due to the international acceptance by health organisations that they provide best available information to guide good quality of care and prevent harmful interventions [15]. This manuscript is a sub-study of an umbrella project examining population-wide overuse and underuse of prescribing for cardiovascular diseases whose protocol was not registered. Estimates of overuse and underuse of anti-hypertensive and cholesterol-lowering medications will be reported elsewhere.

### Inclusion criteria

The target population (P) was adult patients in any setting (e.g. hospital, primary care, or community). Eligible interventions (I) were prescribing or deprescribing of antithrombotics (i.e. anticoagulants or antiplatelet agents) administered by any route for primary or secondary prevention of either thromboembolic events or management of bleeding complications. Comparators (C) were not always included as included studies were trials and beyond. Outcome (O) of interest was objectively measured adherence against explicitly stated and referenced guidelines whether international, national or regional. Articles were eligible if they reported at least one of the indicators (overuse or underuse) where objective outcomes were measured in quantifiable ways. In intervention studies that aimed to increase guideline adherence, we did not limit to those who used STOPP/START criteria but accepted any method of investigating the outcome in relation to the guideline, and only reported baseline outcome data that reflected usual practice. Eligible quantitative study (S) designs included: retrospective record reviews, prospective cohorts, interventional studies whether samples were randomly assigned or not, time series, before-after studies, cross-sectional surveys or audits, and secondary analyses of disease registries.

### Exclusion criteria

Excluded were articles using a guideline non-adherence definition which combined both overuse and underuse in a single estimate; having self-reported measures (e.g. clinician surveys of perceived guideline adherence, patient- reported doctor recommendations); failing to mention reference guideline or using internally developed but unpublished guideline. Conference abstracts without sufficient information to assess risk of bias, case studies, study protocols without data, qualitative consultations of perceived inappropriateness, commentaries, and editorial pieces were ineligible.

### Search strategy

Our search strategy targeted published English language literature from Medline and EMBASE databases from January 2000 to May 2021 to reflect recent and current practice involving new generation oral anticoagulants. The umbrella search strategy included the terms ‘guideline,’ ‘adherence’ and ‘prescribing’ among others (details in Supplement 1, Table S1.1), was designed jointly by the team based on clinical experience, and subsequently refined by our information specialist JC using polyglot tool [16], word frequency counter, and de-duplicator from the internally developed and tested systematic review accelerator [17].

### Screening and data extraction

Subsets of title, abstract and full text screening was conducted by paired authors independently (LC, MC, OB, LH, MO, TH) with discordances resolved by discussion using the disputatron tool [17]. A purpose-built data extraction template including author, publication year, country, sample size, study design, setting, target conditions/guideline topic, and study population was used. Estimates of overuse and underuse were extracted by paired authors (LC, MC, LH, OB) either from the text or tables, and the accuracy of data extraction double checked and confirmed by the statistician (MJ) who subsequently built forest plots. Drivers of overuse and underuse as well as potential solutions were extracted by the lead author (MC) from the discussion sections of eligible papers to enhance the context of our discussion.

### Outcome definitions

Our primary outcome was the estimate of percentage overuse or underuse or equivalent terms (excess, under-adherence, underutilisation, overprescribing, non-compliance -if stated the direction, etc). Overuse was defined as follows: prescribing when not clinically indicated (e.g., CHADS_2_ score = 0), excess dose/duration, or inappropriate or unnecessary administration route (e.g., IV or subcutaneous medication when an oral alternative would have sufficed). Underuse was defined as non-prescribing when recommended by the guideline such as CHADS_2_ score ≥ 2, or lower than recommended dose or shorter duration according to the guidelines used as gold standard. Outcomes were expressed as a percentage of patients managed with or without anti-thrombotics out of eligible patients for prescribing or non-prescribing.

### Data synthesis

Estimates of overuse and underuse are presented, where possible, as reported by the published authors, predominantly as mean percentages with 95% confidence intervals. When this information was not reported but absolute numbers for numerators and denominators were presented in the text or tables, we calculated mean percentages with 95% confidence intervals. If the study included an intervention, only pre-intervention data were reported. Given the heterogeneity of study designs, populations, guidelines and outcome measurements, subgroup analysis by setting, region or clinical indication was limited to narrative description. No attempt was made to impute missing data or conduct sensitivity analyses. Forest plots were produced separately for overuse and underuse for ease of visualization, but no meta-analysis was undertaken. Reported findings followed the synthesis without meta-analysis guideline [18].

### Risk of bias

Given the anticipated inclusion of trials, cross-sectional and cohort studies, risk of bias was based on Hoy’s published 10-item tool for assessing prevalence studies [19]. We modified the version by using the checklist with the exception of items 5 and 9 which were not directly relevant to our research question.

Paired reviewers (LC, MC, OB, LH, MO) independently assessed each study completing the template with six key questions for all eligible study designs (Supplement 1, Table S1.2). Two senior authors (TH, MC) assisted in resolving risk of bias discrepancies. Trained academics not members of the overuse research team (LA, MB, EL in acknowledgment) also assisted occasionally in the resolution of risk of bias if agreement could not be achieved and senior reviewers were unavailable.

## Results

### Study selection

**Figure 1:**
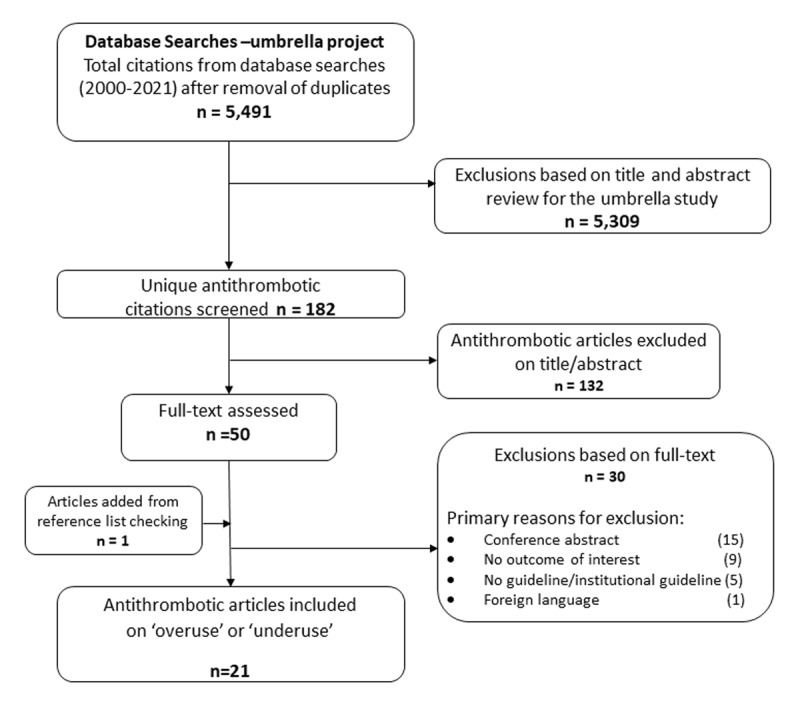
PRISMA diagram for selection of eligible studies.

Twenty-one contemporary studies (published between 2008 and 2021) met the inclusion criteria. They were conducted in 14 countries across four continents (Europe/UK, n = 11, North America, n = 5, Australia n = 3, and Asia, n = 2) and involved a total of 167,287 participants. Figure 1, the PRISMA diagram shows details of the selection process).

### Study characteristics

Study designs included 14 retrospective record reviews [20,21,22,23,24,25,26,27,28,29,30,31,32,33], 6 cohort studies [34,35,36,37,38,39] and 1 a case-control [40] (see Table 1). Four studies focused on overuse, 5on underuse, and 12 on both. Information derived predominantly (n = 18) from hospitalized patients or disease registries, with only three studies in primary care. The clinical context included preventive use in atrial fibrillation (AF, n = 8), perioperative bridging (n = 5) and treatment of various conditions (stroke, venous thrombosis, myocardial infarction, excess anticoagulation, trauma management; n = 8).

**Table 1:** Overuse and underuse study characteristics by study design and setting (N = 21).


AUTHORS AND PUBLICATION YEAR	COUNTRY	SAMPLE SIZE	STUDY DESIGN		SETTING			TARGET CONDITIONS/CLINICAL ACTIVITY	TARGET TOPIC	
		
			RETROSP REVIEW	COHORT/C-C	HOSPITALISED	PRIMARY CARE	COMM/OUTPAT		OVERUSE	UNDERUSE

								**Overuse**		

Moesker et al., 2019 [21]	NL	256	✓		H			Reviewing the bridging anticoagulation policy for acute or elective surgical procedures	✓	✓

Wong et al, 2015 [24]	Australia	19,613	✓		H			Anticoagulation for non-valvular AF in high and low-risk patients	✓	✓

Admassie et al, 2017 [20]	Australia	625	✓		H			Anticoagulants in patients at risk of stroke from non-valvular AF	✓	✓

Wertheimer et al., 2019 [23]	Australia	200	✓		H			Anticoagulants for valvular and non-valvular AF	✓	✓

Vesa et al., 2020 [22]	Romania	784	✓		H			Antithrombotics in non-valvular AF	✓	✓

Gorczyca et al., 2020 [25]	Poland	1,236	✓		H			Prophylactic antithrombotic therapy among patients with AF	✓	✓

Steib et al, 2014 [29]	France	394	✓		H			Perioperative Vit K antagonists	✓	✓

Manoucheri et al., Fallahi, 2015 [27]	Iran	472	✓		H			Antithrombotic agents for prophylaxis and treatment of VTE	✓	✓

Khatib et al., 2020 [26]	USA	13,677	✓		H			Post-discharge home-based antithrombotic therapy for VTE	✓	

Rosignol et al, 2019 [28]	France	145	✓		H			Management of traumatic bleeding in patients with injury severity score of >16		✓

Waechter et al., 2020 [30]	Germany	373	✓		H			Anticoagulants for persistent AF and mitral valve repair patients undergoing TMVR		✓

Boivin-Proulx et al., 2020 [34]	Canada	459		Coh	H			Antithrombotics for AF on patients undergoing percutaneous coronary intervention with coronary stenting		✓

Giustozzi, M 2020 [36]	Italy	155		Coh	H			Antithrombotics for stroke/Transient Ischaemic attack in patients known to have AF before admission		✓

Jortveit et al., 2019 [37]	Norway	47,204		Coh	H			Anticoagulants for AF in patients with myocardial infarction who were in the registry		✓

Uzieblo-Zyczowska et al., 2021 [39]	Poland	359		Coh	H			Antithrombotics for AF on patients undergoing percutaneous coronary intervention	✓	✓

Moerlie et al., 2020 [38]	NL	411		Coh	H			Dual Antithrombotics for multiple conditions in hospital inpatients	✓	

Devine et al, 2009 [35]	USA	417		Coh	H		O	Management of excess warfarin anticoagulation	✓	✓

Laughenburger et al, 2015 [31]	USA	70,498	✓		H		C	Anticoagulants first prescription for patients diagnosed with AF	✓	

Miyazawa et al., 2019 [33]	Japan & UK	4,239 2,259	✓ ✓			P	C	Antithrombotics for stroke prevention in AF using 2 registries	✓	✓

Le Blanc et al., 2020 [32]	Canada	1,681	✓			P		Anticoagulants for permanent, paroxysmal or persistent non-valvular AF	✓	

Vanbeseleare et al, 2016 [40]	Belgium	1,830		C-C		P		Anticoagulants for treatment of AF within 6 months of diagnosis	✓	✓


C = Community setting; O = Outpatients; CC = Case-Control; Coh = Cohort; NL = The Netherlands.

### Risk of bias assessment

**Figure 2:**
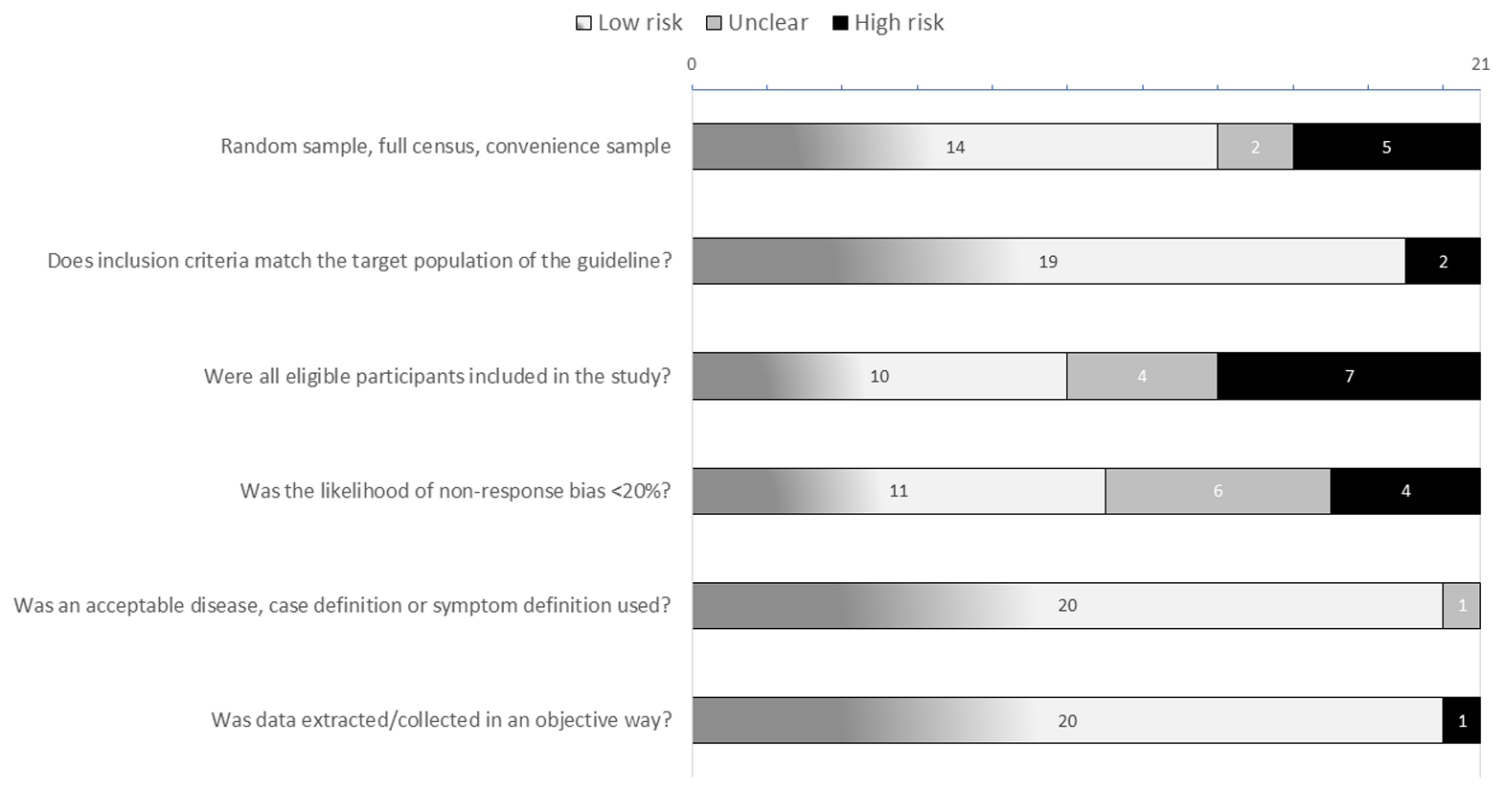
Risk of bias across the included studies (N = 21).

Across the studies, risk of bias was generally low in terms of the inclusion criteria matching the guideline’s target population, provision of a clear and acceptable case definition, and objective data extraction methods (Figure 2).

However, in some studies, there was a high risk of selection bias from use of either convenience sub-samples, or unclearly presented selection methods where not all potentially eligible patients were included, incomplete response or final outcome ascertainment of ≥20%, and non-inclusion of full census, consecutive patients, or random subject selection. Six studies did not report the extent of missing follow-up data. No selective reporting was observed as most studies measured a single primary outcome.

## Synthesis of Results

### Overuse estimates

**Figure 3:**
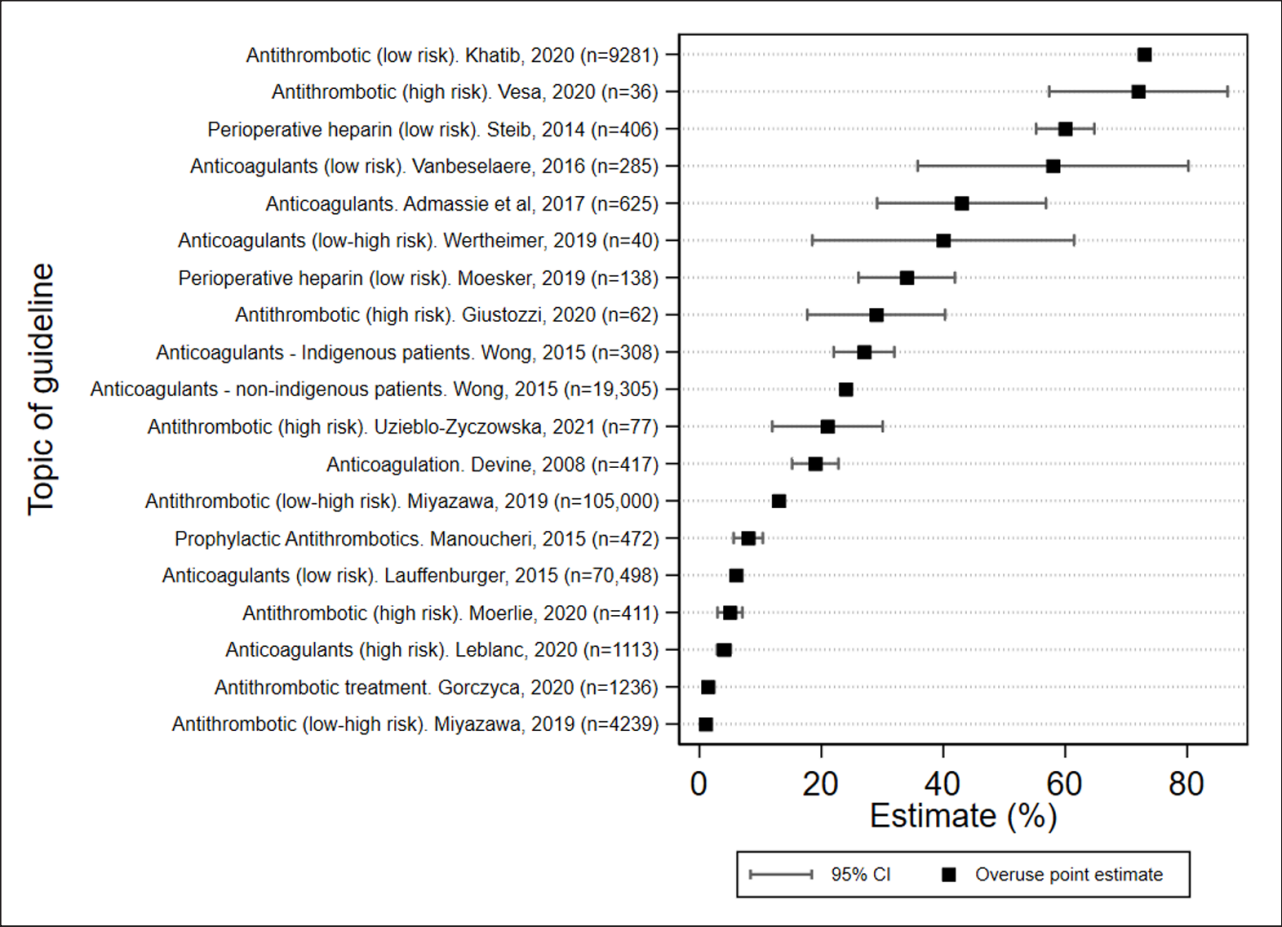
Estimates of overuse of antithrombotic interventions across clinical settings (N = 17 studies).

Large variation in the rates of antithrombotic overuse was observed across geographic regions with studies from Asia reporting the lowest levels of overuse (1.3–7.6%) [27,33], followed by Australia (24.1–42.9%) [20,23,24], and Europe (1.4–72.0%) [21,25,28,29,30,33,36,37,38,39,40], through to the highest variation in North America (6.6–73.0%) [26,31,32,34,35]. No clear time trend of increasing or decreasing overuse was observed over the period of the studies (2008–2020). Estimates in 10 (of 17) studies had narrow confidence intervals due to moderate to large sample sizes (Figure 3).

The most commonly reported indication of antithrombotic overuse was in prophylaxis for people with atrial fibrillation (AF) at low risk of stroke or other thrombotic events. As shown in Figure 3, overuse ranged from a low of 1.3% for stroke prevention in a Japanese cohort with AF [33] and a 1.4% in a Polish study [25], to a high of 72.2% prophylactic use in non-valvular AF in Romania [22]. Overall, median overuse for this indication was 24% (IQR 5.0%–42%).

The retrospective studies of AF patients conducted in primary care or using community registries tended to report the lowest levels of overuse in this collection, such as 1.3% in Japan [33], 4.0% in Canada [32], 6% in the largest sample (n = 70,498) in the US [31], and 13.1% in the UK [33]. The exception was a case-control study of AF patients within six months of diagnosis in multiple Belgian general practices which found overprescribing of 57.9% [40].

There was a wide range in overuse in venous thromboembolism (VTE) treatment in hospitals, from 7.6% in a study in Iran [27] to 73% in a US study of patients admitted for VTE treatment contrary to guideline recommendation to manage them as ambulatory patients [26].

Among the three studies of perioperative bridging anticoagulation reporting overuse, estimates varied from 21% in a Polish cohort of patients with AF undergoing percutaneous coronary intervention [39], through to 34% in a retrospective review of Dutch patients receiving Vit K antagonists before acute or elective surgeries [21], and up to 59.6% of French patients in people undergoing elective or emergency procedures [29]. A North American cohort study of patients presenting with prolonged prothrombin time INR ≥4.5 reported as 22.3% due to unjustified administration route or excess dose [35]. Median overuse across these studies was 34% (IQR 27.5 – 46.8%).

### Underuse Estimates

**Figure 4:**
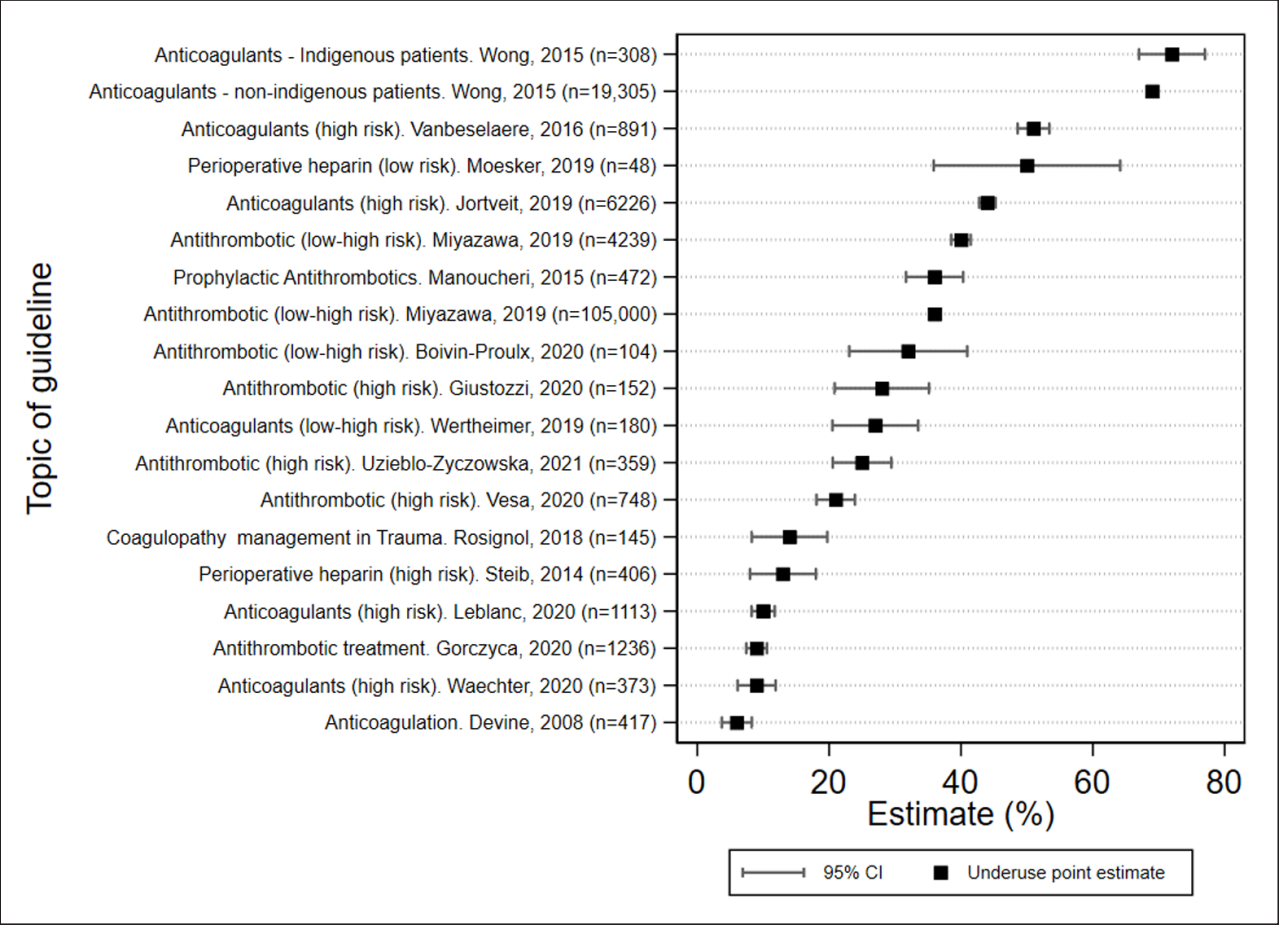
Estimates of underuse of antithrombotic interventions across clinical settings (N = 17 studies).

Variation in underuse of appropriate anticoagulation was observed across clinical indications with estimates of greater than 30% in 6 of the 17 studies (Figure 4) [21,24,27,33,37,40]. The highest rate of underuse was in adults with AF at high risk of stroke who were not prescribed anticoagulant therapies in a tertiary referral hospital, with similar underuse rates in both Indigenous Australians (72%) and non-Indigenous patients (69%) [24].

Substantial underuse of prophylactic anticoagulants in AF was also reported (51.4%) by a Belgian case-control study in primary care [40], as well as the AF community registries in Japan (40%) [33] and the UK (36%) [33]. Moderate estimates of prophylactic underprescribing (27% and 21%) were derived from retrospective record reviews in Australia and Romania respectively [22,23]. The lowest estimates of underuse for this indication were found by two retrospective chart audits in Poland hospitals (9.0%) [25] and primary care in Canada (10%) [32]. Overall, median prophylactic underuse in AF was estimated at 36.0% (IQR 21.0–51.4%).

Underuse of VTE treatment for people with clinical indication was reported in over a third (36%) of Iranian patients admitted to a teaching hospital [27]. Two recent hospital studies reported the extent of antithrombotic undertreatment for management of transient ischaemic attack (28%) or acute myocardial infarction (44%) in Italy and Norway respectively [37,41].

Underuse of bridging coagulation in the perioperative period was found to be present in 33.0% cases in a Canadian hospital cohort [34] and wide variation between 9.0 and 50.0% was found in Europe across three retrospective hospital audits [21,29,30] and one hospital cohort [39]. A US cohort of patients presenting with excess coagulation found 6.2% under-administration of vitamin K [35], and a retrospective analysis of management or traumatic injuries to prevent coagulopathy and thromboembolic events in a French hospital reported 14.3% underprescribing of thrombolytic agents [28]. Median underuse in perioperative bridging across the five studies was 25.0% (IQR 13.1 – 32.0%). While no time trend was visually apparent, underuse estimates from five retrospective chart reviews [22,25,26,30,32] and four cohort studies [34,36,38,39] since 2020 in Europe and North America have remained below 33% (9–32%) by comparison with 36%–72% reported between 2015 and 2019, but we did not formally test this.

### Determinants of guideline non-adherence (underuse and overuse)

The reasons for underuse were generally not mentioned in our identified articles. Among the one third of included articles reporting reasons, it was clear that justifications for underuse were largely clinician-driven by fear of complications or insufficient knowledge of specific guidelines. By contrast, most of the determinants of overuse when reported were system related, with minority attributed to patient preference (Table 2).

**Table 2:** Clinician, patient and system determinants of overuse and underuse.


REASON FOR OVERUSE ^[REFERENCE #]^		REASON FOR UNDERUSE ^[REFERENCE #]^	

P	Low-risk patients with genuine indication for anticoagulants for other non-AF conditions [40]	C	Fear of patient bleeding complication; overestimation of risk over benefits [20,22,24,32,36]

P	Low-risk patient preference to minimize risk of stroke [23]	C	CHA2DS2-VASc risk scores not documented or incorrect [23]

		C	Clinician lack of knowledge of the disease [27] Clinician’s lack of awareness of stroke risk from non-use of the combined CHA2DS2-VASc score and bleeding (HAS-BLED) score [22]

S	Update in guidelines in some countries no longer recommending antiplatelet agents in AF make others appear overprescribing [20,33]	C	GP perceived risk of bleeding if history of peptic ulcer or tumour [40]

S	Absence of a national guideline [20]	C	Doctor perceived lower thromboembolic risk in women than in men [20,40]

S	Evolution in risk prediction and wide availability of direct OA [24]	C	Older age a barrier to start OA [25,32] due to clinician’s perceived risk of bleeding [20,40]

S	Patient comorbidities, lack of social support or insurance status as incentive for in-hospital management [26]	C	Falls risk reduces clinician inclination to prescribe [20,23,40]

S	Aggressive promotion by pharmaceutical companies [20]	C	Lower inclination to prescribe in dementia, frailty syndrome [25], known poor patient compliance [40]

S	Lack of registry information on discontinuation at subsequent time points [33]	P	Patients’ unwillingness to receive prescription and non-adherence after prescription [24,33]

C	Low clinician familiarity with or adoption of risk stratification methods [20,26] or guidelines [24,27]	P	Documented contraindication: scheduled surgical procedure, active bleeding, reduce glomerular filtration, alcoholism [23,25,32,36]


AF = atrial fibrillation; OA = oral anticoagulants; C = clinician reason, P = patient reason; S = system determinant.

None of the studies specifically examined or reported the influence of patient preferences for anticoagulant type. However, a study comparing patient and clinician preferences for anticoagulation found difference in preferences: the minimum number of strokes that needed to be prevented to justify anticoagulation was significantly lower for patients than for physicians (1.8 versus 2.5 per 100 patient years), and the maximum number of excess bleeds that was acceptable was significantly higher for patients than for physicians [42]. No study reported using decision aids to inform the risk of bleeding events versus the benefits of stroke prevention.

### Potential solutions

Brief solutions listed in some included studies without further elaboration and other relevant recommendation from different specialties are shown in Table 3. Addressing overuse, underuse and practice variation is likely to need strategies at the patient, clinician and system levels. Some voluntary or mandatory adoption policies may play a role in reducing variation but the proliferation of guidelines from different groups and updated recommendations for treatment across countries such as in the case of antithrombotics for AF may generate confusion for clinicians managing different at-risk populations [43].

**Table 3:** Proposed solutions for overuse and underuse from included studies and other literature.


**To reduce overuse**

Integration of pharmacists in post-discharge follow-up to cease time-limited medication when no longer indicated [38]

Training of and alerts for high-volume prescribers [44]

Decision support tools [45]

Public awareness campaigns [4]

Health literacy programs on overdiagnosis to reduce healthcare expectations [46]

**To reduce underuse**

Patient education on long-term benefits of anticoagulation and on enhancing self-care [22]

Clinician education on calculating/interpreting stroke risk and bleeding risk [22]

Clinician education on old age, comorbidities and dementia not being contraindications for anticoagulants [47]

**To reduce practice variations**

Development of national guidelines, and clinician education on customizing treatments to different risk levels[20] including reversal of overtreatment [48]

Wider availability of direct-acting oral anticoagulants to replace vitamin K antagonists which are more prone to mis-prescribing [20]

Quality improvement initiatives with group or individual feedback [49]

Policies mandating the use of protocols for healthcare delivery [50]

Practice incentives to fast-track evidence uptake [51]


It can be argued that guideline non-adherence can be caused by the knowledge that some guidelines are flawed if based on expert consensus rather than on high level of evidence [52]; or even when trial evidence exists the recommendations only apply to a limited type of patients [53]. The single-disease guideline focus may also contribute to non-adherence as they may not apply to patients with multiple comorbidities [54]. In sum, estimates of overuse and underuse based on comparison against static practice guidelines with data collections not linked to reasons for clinical decisions can only provide an indication, and may be prone to error rates in both directions.

## Discussion

The findings of this review strongly suggest widespread overuse and underuse of antithrombotic agents worldwide by summarising estimates of non-adherence to guidelines for the management of multiple conditions. Our findings highlight antithrombotic prescribing variation across and within health systems, including persistent overuse (1.3–73.0%, median 24.1%, IQR 6.8–41.5) and underuse (6.2–72.0%, median 28.0%, IQR 13.8–42.0) across hospitals, primary care and community settings for over a decade (2008–2021). Overuse was mostly reported among AF patients for stroke prevention or for perioperative bridging. Underuse was mostly reported for bridging coagulation in the perioperative period. Most included studies had low risk of bias for many of the domains assessed, such as random samples or full patient census, clear case definitions, and objective measures related to published guidelines.

This review found that 1 in 4 patients with AF and 1 in 3 perioperative patients are overtreated, and that 1 in 3 AF patients and 1 in 4 perioperative patients are undertreated. This strongly indicates the need to ascertain the root causes and implement strategies to reduce risk of complications and preventable healthcare costs. The applicability of results is further evident in potential benefit of guideline adherence to improve patient outcomes by reducing adverse events, hospitalisations and mortality. However, only a handful of eligible studies examined outcomes beyond compliance with guidelines [26,33,36]. This is most likely due to the retrospective or cross-sectional nature of most designs and the lack of linkage beyond individual registries or institutions.

Our approach to use any guideline adopted by the research teams in their country or setting reflects real-life practice. Association with clinician’s decisions for deviation from guidelines would have added reliability to the estimates but unfortunately automation of the text fields for non-adherence reasons within the core variables of the electronic medical record was not commonplace. Despite some guidelines differing across studies, this measurement (i.e. % non-adherence in either direction in relation to relevant guideline) facilitates the comparative reporting of a standard outcome to inform public health policies and variation in practice.

### Comparisons with other published studies

Other literature reviews that were either non-systematic or conducted on single medications reported much higher rates (40.0%–85.6%) of under-prescribing of anticoagulants for AF internationally [9,55]. Similarly, a large cohort study conducted in 28 countries [56] reported variation in prevalence of initial therapy for and undertreatment of VTE at 46.5% during pregnancy. This study did not meet our eligibility criteria because the comparator for four continents was the USA guidelines, which renders some of the overuse or underuse estimates invalid for other national contexts in light of different risk stratification approaches and local management guidelines [57].

While a previous review reported increasing overuse of multiple practices over the years [58], the estimates in our study did not indicate a clear trend in guideline adherence improvement or worsening overtime, but we did not formally examine this. These mixed results across all years could be due to clinician’s deliberate decisions (i.e. discretionary care) [58], availability of medications and tests in different health systems, as well as changes in patient attitudes or clinician awareness of guideline updates [59].

Such inconclusive results of non-guideline concordant use of prescribing must be interpreted with caution as some studies spanned several years and guideline recommendations changed in that period, where uptake in routine care is known to take years. This may have led to re/misclassification of guideline adherence and non-adherence and made previously accepted practices non-concordant in more recent times. Further, some alarming overestimates were derived from small denominators, and other small estimates emerged from large registries or hospital chart reviews.

### Limitations

Our search strategy was applied to only two major databases in English language and some studies may have been missed as a result. However, the search strategy underwent extensive review and iteration to ensure search output efficiency and accuracy. Our search strategy did not include the terms ‘omission’ or ‘commission’ that might have been used by others to indicate underuse. We also excluded studies using local or single institution guidelines and studies of single treatments or single conditions as we were interested in population-wide impact of guideline non-adherence of recognised guidelines. Our decision may have resulted in an underestimate; however, it makes these findings more generalisable at population level. Heterogeneity of study methods, sources and outcome definitions precluded meta-analysis. We chose guidelines as a generally accepted reference of appropriateness. Yet, we acknowledge that not all guidelines have been developed through a comprehensive review of the scientific evidence [13] and some of the recommendations do not apply to patients of all ages, with multiple underlying conditions [60], allow for clinical flexibility, or consider all organisational constraints in all settings. Further, recommendations in clinical guidelines also change overtime, and their publication can be delayed, leading to true adherence estimates not being reflected [61]. While guideline adherence was objectively measured from available data sources, the authors of included papers did not generally report how clinical decisions were made or how individual exemptions were taken into account. Since the data were largely not linked to reasons for clinical decisions, it cannot be unequivocally claimed that all reported cases of non-adherence were true overuse or underuse if justifiable exemptions existed. In sum, level of adherence to guidelines can only be considered a best surrogate indicator of medication safety [62].

### Implications for practice and research

Overprescribing in the absence of a clinical indication, hospitalising for anticoagulation management when ambulatory treatment is considered safe, and underprescribing for patients with high-risk profile identified in this review can expose patients to further risk of complications such as bleeding or preventable thromboembolic events. While getting the balance right between too much and not enough takes expertise, guidelines are a best available reference point. Importantly, many factors may have contributed to the wide variations in practice found in this review: use of a reference guideline from a different country to the study setting; differences in availability of medicine types across health systems; co-payment schemes influencing prescribing patterns or patient preference; true unawareness of the guideline by prescribers; clinician’s personal biases, preferences or caution based on previous experience with adverse events; and the actual methodology of individual studies.

Our findings highlight some critical issues for attention of future guideline developers in this area, such as reporting of explicit exemptions. We were unable to find examples of introduction of machine learning algorithms for retrospective assessments of non-adherence using clinical exemptions in decision-making. The usefulness of integrated pharmacy and health service records in predicting future patient adherence [63] or estimating individualised optimal heparin doses [64] is promising. There are also high hopes for machine learning to contribute identification of statistical patterns of prescribing quality [65] but their effectiveness in identifying meaningful and credible conclusion on clinician’s guideline concordance and decision parameters are still under investigation [66]. So far prospective attempts to encourage and enhance adherence to guideline targets in cardiovascular disease management through computerised clinical decision making tools have not achieved substantial gains [67].

A gap identified in the included articles of this review were the impact of guideline non-adherence on health outcomes for patients, and the possible impact of using decision aids before antithrombotics prescribing on patient behaviour. Both were beyond scope, and future qualitative research could assist in improving understanding of the reasons for overuse or underuse by different stakeholders. Ascertainment of the reasons for unwarranted variations – whether clinician or patient driven – is important to inform the development of tailored de-implementation interventions. Future studies could investigate the economic impact of over/underprescribing on health service utilisation, and the avoidable cost of over-treatment and propose a lower/acceptable limit of what is ‘acceptable/unavoidable healthcare resource waste.’

## Conclusions

Substantial efforts have been made by multiple clinical bodies across countries to produce guidelines for management of thromboembolic and bleeding risks. Despite this, overuse and underuse of antithrombotic agents continues to be a problem across health systems worldwide. Clinicians’ previous experience, challenges in keeping up-to-date with evolving guidelines, and their patients’ preferences are barriers to adherence. The need to understand clinicians’ motivations to adopt or deviate from guidelines is an ongoing research goal to inform interventions to halt overuse and underuse practices.

Reliable monitoring of adherence or non-adherence to practice guidelines should extend to routine linkage of electronic medical records across the screening-diagnosis-treatment cycle. Further, to make indicators clearer on research and health service information systems, supplementation with clinical indications for use or reason for deviation from recommendations in the electronic medical record, and machine learning techniques to generate automated, sophisticated algorithms could more clearly distinguish between low-value care practices and acceptable exemptions from the guidelines in retrospective studies. Until supplementary clinical information for deviations from guidelines, and linkage with patient outcomes becomes routinely available, it is not possible to make recommendations on thresholds for decision-making on minimal level of justifiable overuse for antithrombotic prescribing.

## Additional File

The additional file for this article can be found as follows:

10.5334/gh.1142.s1 Supplementary Material. Supplement Tables S1.1–1.2 and S2.1.

## Data Availability

All data relevant to the study are included in the article or uploaded as online supplementary information. For any further queries please contact the corresponding author.

## Abbreviations

AF, atrial fibrillation; CHADS, score to predict risk of stroke (congestive heart failure, hypertension, age ≥75 years, diabetes mellitus, prior stroke or TIA or thromboembolism); INR, international normalized ratio; IQR, interquartile range; VTE, venous thromboembolism.
