# Supplementary material for: Guideline Adherence As An Indicator of the Extent of Antithrombotic Overuse and Underuse: A Systematic Review
Source: Glob Heart. 2022 Aug 12;17(1):55. doi: 10.5334/gh.1142 (PMC9374022; doi:10.5334/gh.1142)
Supplement: Supplementary Material. — Supplement Tables S1.1–1.2 and S2.1. [file gh-17-1-1142-s1.pdf]

Supplementary Material

1 Supplementary Tables

Supplement 1. Table S1.1 Search strategy

**Ovid MEDLINE Search**
((exp Guideline Adherence/ OR<br><br>((Guideline.ti,ab. OR Guidelines.ti,ab. OR National recommendations.ti,ab.) adj8 (Comparison.ti,ab. OR Adherence.ti,ab. OR Adherent.ti,ab. OR Concordant.ti,ab. OR Concordance.ti,ab. OR Discordant.ti,ab. OR Compliance.ti,ab. OR Deviation.ti,ab. OR Deviations.ti,ab. OR overuse.ti,ab. OR underuse.ti,ab.)))<br><br>AND<br><br>(Prescribing.ti,ab. OR Prescription.ti,ab. OR Prescriptions.ti,ab. OR Treat.ti,ab. OR Treating.ti,ab. OR Treatment.ti,ab. OR Treatments.ti,ab. OR Intervention.ti,ab. OR Interventions.ti,ab. OR Procedure.ti,ab. OR Procedures.ti,ab.)<br><br>AND<br><br>(Rate.ti,ab. OR Rates.ti,ab. OR Frequent.ti,ab. OR Frequency.ti,ab. OR Treated.ti,ab. OR Prescribed.ti,ab. OR Management.ti,ab. OR Overuse.ti,ab. OR Underuse.ti,ab.)<br><br>AND<br><br>(exp Practice Patterns, Physicians'/ OR exp Episode of Care/ OR exp Disease Management/ OR Practice.ti,ab. OR Practices.ti,ab. OR Practitioners.ti,ab. OR Physicians.ti,ab. OR Physician.ti,ab. OR Pattern.ti,ab.)<br><br>AND<br><br>("Practice Guidelines as Topic"/ OR exp case-control studies/ OR exp Cohort Studies/ OR Cohort.ti,ab. OR Observational.ti,ab. OR Prospective.ti,ab. OR Retrospective.ti,ab. OR Cross sectional.ti,ab. OR exp Cross-Sectional Studies/ OR Analyzed.ti,ab. OR Analysed.ti,ab. OR Investigated.ti,ab. OR (statistics and numerical data).fx.)<br><br>NOT<br><br>("Surveys and Questionnaires"/ OR Survey.ti. OR Surveys.ti. OR Surveyed.ti. OR Questionnaire.ti. OR Questionnaires.ti.)<br><br>)<br><br>AND<br><br>(Guideline.ti. OR Guidelines.ti. OR National recommendations.ti. OR Comparison.ti. OR Adherence.ti. OR Adherent.ti. OR Concordant.ti. OR Concordance.ti. OR Discordant.ti. OR Compliance.ti. OR Deviation.ti. OR Deviations.ti. OR overuse.ti. OR underuse.ti. OR Prescribing.ti. OR Prescription.ti. OR Prescriptions.ti. OR Treat.ti. OR Treating.ti. OR Treatment.ti. OR Treatments.ti. OR Intervention.ti. OR Interventions.ti. OR Procedure.ti. OR Procedures.ti. OR Rate.ti. OR Rates.ti. OR Frequent.ti. OR Frequency.ti. OR Treated.ti. OR Prescribed.ti. OR Management.ti. OR Practice.ti. OR Practices.ti. OR Practitioners.ti. OR Physicians.ti. OR Physician.ti. OR Pattern.ti.)

**Embase search**
(<br><br>('protocol compliance'/exp/mj OR<br><br>((Guideline OR Guidelines OR "National recommendations") NEAR/8 (Comparison OR Adherence OR Adherent OR Concordant OR Concordance OR Discordant OR Compliance OR Deviation OR Deviations OR Overuse OR Underuse)):ti,ab)<br><br>AND<br><br>(Prescribing:ti,ab OR Prescription:ti,ab OR Prescriptions:ti,ab OR Treat:ti,ab OR Treating:ti,ab OR Treatment:ti,ab OR Treatments:ti,ab OR Intervention:ti,ab OR Interventions:ti,ab OR Procedure:ti,ab OR Procedures:ti,ab)<br><br>AND<br><br>(Rate:ti,ab OR Rates:ti,ab OR Frequent:ti,ab OR Frequency:ti,ab OR Treated:ti,ab OR Prescribed:ti,ab OR Management:ti,ab)<br><br>AND<br><br>('clinical practice'/exp/mj OR 'patient care'/exp/mj OR 'disease management'/exp/mj OR Practice:ti,ab OR Practices:ti,ab OR Practitioners:ti,ab OR Physicians:ti,ab OR Physician:ti,ab OR Pattern:ti,ab)<br><br>AND<br><br>('practice guideline'/de OR 'case control study'/exp OR 'cohort analysis'/exp OR Cohort:ti,ab OR Observational:ti,ab OR Prospective:ti,ab OR Retrospective:ti,ab OR "Cross sectional":ti,ab OR 'cross-sectional study'/exp OR Analyzed:ti,ab OR Analysed:ti,ab OR Investigated:ti,ab)<br><br>NOT<br><br>('questionnaire'/de OR Survey:ti OR Surveys:ti OR Surveyed:ti OR Questionnaire:ti OR Questionnaires:ti OR Review:it OR "systematic review":ti)<br><br>)<br><br>AND<br><br>(Guideline:ti OR Guidelines:ti OR "National recommendations":ti OR Comparison:ti OR Adherence:ti OR Adherent:ti OR Concordant:ti OR Concordance:ti OR Discordant:ti OR Compliance:ti OR Deviation:ti OR Deviations:ti OR Overuse.ti. OR Underuse.ti. OR Prescribing:ti OR Prescription:ti OR Prescriptions:ti OR Treat:ti OR Treating:ti OR Treatment:ti OR Treatments:ti OR Intervention:ti OR Interventions:ti OR Procedure:ti OR Procedures:ti OR Rate:ti OR Rates:ti OR Frequent:ti OR Frequency:ti OR Treated:ti OR Prescribed:ti OR Management:ti OR Practice:ti OR Practices:ti OR Practitioners:ti OR Physicians:ti OR Physician:ti OR Pattern:ti)

Supplement 1. Table S1.2 Risk of bias key questions

1. Was the study sample randomly selected, a census or full population?	Yes, No, Unclear
2. Did inclusion criteria match the target population of the guideline?	Yes, No, Unclear
3. Were all eligible participants included in the study?	Yes, No, Unclear
4. Was the likelihood of non-response bias low (<20%)?	Yes, No, Unclear
5. Was an acceptable disease, case definition or symptom definition used?	Yes, No, Unclear
6. Was data extracted or collected in an objective way?	Yes, No, Unclear

Supplement 2 Table S2.1. Detailed reasons for exclusion of full texts

#	Full reference in alphabetical order (cut n paste from EndNote Preview)	Pre-coded reason
1	Cryder, B., & Glosner, S. (2020). Anticoagulation quality assessment and risk evaluation in patients with nonvalvular atrial fibrillation (NVAF) at a midwest internal medicine practice. JACCP Journal of the American College of Clinical Pharmacy, 3(8), 1588. doi:10.1002/jac5.1351	Conference Abstract with insufficient data
2	Dakhil, Z. A., Farhan, H. A., & Hasan, H. S. (2020). Oral Anticoagulation in Heart Failure with Atrial Fibrillation: Are We Taking the Right Steps Towards Guidelines? Journal of Cardiac Failure, 26(10), S94. doi:10.1016/j.cardfail.2020.09.275	Conference Abstract with insufficient data
3	Denis, L., Fodil, M., Sterpu, R., Ricome, N., Drouot, S., Rieutord, A., . . . Chaumais, M. C. (2019). Guidelines compliance of oral anticoagulants in geriatrics ordered and monitoring in a French hospital group. International Journal of Clinical Pharmacy, 41(1), 340. doi:10.1007/s11096-018-0759-9	Conference Abstract with insufficient data
4	Han, H., Sippola, E., Chen, W., Morgan, S., Renner, E., Ruff, A., . . . Barnes, G. D. (2020). Implementation of Pharmacist-led Antithrombotic Medication Management for Elective Endoscopic Procedures. Circulation, 142(SUPPL 3). doi:10.1161/circ.142.suppl_3.14379	Conference Abstract with insufficient data
5	Lam, S., Pepperell, D., & P'ng, S. (2019). Perioperative management of direct oral anticoagulants: A single centre observational study. Research and Practice in Thrombosis and Haemostasis, 3, 731-732. doi:10.1002/rth2.12229	Conference Abstract with insufficient data
6	Marinelli, S., Zoli, M., Calogero, P., Lunardelli, M. L., & Bastagli, L. (2019). Adherence to the 2016-2018 ESC guidelines for stroke prevention in atrial fibrillation: An Italian field practice in a cohort of geriatric patients. European Heart Journal, 40, 1504. doi:10.1093/eurheartj/ehz748.0870	Conference Abstract with insufficient data
7	Mustapha, M., Zainal, H., Tangiisuran, B., Harun, S. N., Looi, I., Sidek, N. N., . . . Aziz, Z. A. (2020). Impact of guidelines non-adherence on mortality among patients managed for acute ischemic stroke. Journal of Pharmaceutical Policy and Practice, 13. doi:10.1186/s40545-019-0201-2	Conference Abstract with insufficient data
8	Rossi, A., Sapio, P. L., Gori, A. M., Ciardetti, N., Vannini, M., Giusti, B., . . . Marcucci, R. (2020). Perioperative Management of oral anticoagulant therapy: From guidelines to real world. Blood Transfusion, 18(SUPPL 4), S426-S427. doi:10.2450/2020.S4	Conference Abstract with insufficient data
9	Tica, O. A., Tica, O., & Popescu, M. I. (2019). Management in atrial fibrillation and heart failure patients. European Heart Journal Cardiovascular Imaging, 20, i80. doi:10.1093/ehjci/jey262	Conference Abstract with insufficient data
10	Van Uden, R. C. A. E. (2020). The effect of a pharmacist intervention on guideline adherence for double and triple antithrombotic therapy in a clinical setting. Pharmacoepidemiology and Drug Safety, 29(SUPPL 3), 206. doi:10.1002/pds.5114	Conference Abstract with insufficient data

#	Full reference in alphabetical order (cut n paste from EndNote Preview)	Pre-coded reason
11	Hung, D., Butler, M., & Campbell, S. (2019). Adherence to Canadian Cardiovascular Society guidelines for prescribing oral anticoagulants to patients with atrial fibrillation in the emergency department. Canadian Journal of Emergency Medicine, 21, S55-S56. doi:10.1017/cem.2019.172	Conference Abstract with insufficient data
12	Vanacker P, Simons S, Verhaeghe A, et al. Concordance between guidelines on perioperative management of noacs and its implementation: Preventable cause of ischemic stroke. International Journal of Stroke 2020; 15: 325. DOI: 10.1177/1747493020963387.	Conference Abstract with insufficient data
13	Zhang, J., Yan, Y. Y., & Zhai, S. D. (2020). A comparative study on the degree of compliance between drug therapy and domestic guidelines for patients with pulmonary embolism based on a registry study. Chinese Journal of New Drugs, 29(9), 1069-1072. Retrieved from https://www.embase.com/search/results?subaction=viewrecord&id=L2007855922&from=export	Conference Abstract with insufficient data
14	Brand, A. R. T., Houben, E., Bezemer, I. D., Visseren, F. J., Bots, M. L., & de Borst, G. J. (2019). Platelet Aggregation Inhibitor Prescription for Peripheral Arterial Disease in the Netherlands. European Journal of Vascular and Endovascular Surgery, 58(6), e573-e574. doi:10.1016/j.ejvs.2019.09.041	Conference Abstract with insufficient data
15	Rhoades, W., Khatib, R., Nitti, K., McDowell, M., Szymialis, R., & Blair, C. (2019). Change in admission rates among patients presenting to emergency departments with VTE and low risk of complications: A retrospective cohort study from 11 community hospitals. Blood, 134. doi:10.1182/blood-2019-126844	Conference Abstract with insufficient data
16	Zyryanov, S. K., Fitilev, S. B., Vozzhaev, A. V., & Shkrebniova, I. I. (2021). Multivariable analysis of primary care physician adherence to guideline-recommended pharmacotherapy of stable coronary artery disease. Rational Pharmacotherapy in Cardiology, 17(1), 29-35. doi:10.20996/1819-6446-2020-16-08	Foreign language
17	Haas, S., Ageno, W., Weitz, J. I., Goldhaber, S. Z., Turpie, A. G. G., Goto, S., . . . Kakkar, A. K. (2019). Anticoagulation therapy patterns for acute treatment of venous thromboembolism in GARFIELD-VTE patients. Journal of Thrombosis & Haemostasis, 17(10), 1694-1706. doi:https://dx.doi.org/10.1111/jth.14548	No guideline referenced as comparator
18	Dreijer AR, Diepstraten J, Leebeek FWG, et al. The effect of hospital-based antithrombotic stewardship on adherence to anticoagulant guidelines. International Journal of Clinical Pharmacy 2019; 41: 691-699. DOI: https://dx.doi.org/10.1007/s11096-019-00834-2.	No guideline referenced as comparator
19	Naebauer, M., Gerth, A., Steinbeck, G., Wegscheider, K., Kirchhof, P., & Breithardt, G. (2019). Prescription of guideline recommended oral anticoagulation and reasons reported for non-use of OAC in patients with atrial fibrillation: Data from the German AFNET-2 Registry. European Heart Journal, 40, 1187. doi:10.1093/eurheartj/ehz748.0644	No guideline referenced as comparator
20	Gerth, A., Nabauer, M., Kirchhof, P., Steinbeck, G., & Breithardt, G. (2019). Prescription of oral anticoagulation (OAC) and reasons reported for non-use of OAC in patients with atrial fibrillation: Data from the german atrial fibrillation network (AFNET) 2 registry.	No guideline referenced as comparator

#	**Full reference in alphabetical order (cut n paste from EndNote Preview)**	**Pre-coded reason**
	Journal of Interventional Cardiac Electrophysiology, 55, S32-S33. doi:10.1007/s10840-019-00559-2	
21	Ghazvinian, R., Elf, J., Löfvendahl, S., Holst, J., & Gottsäter, A. (2020). Outpatient Treatment in Low-Risk Pulmonary Embolism Patients Receiving Direct Acting Oral Anticoagulants Is Associated With Cost Savings. Clinical and Applied Thrombosis/Hemostasis, 26. doi:10.1177/1076029620937352	No guideline referenced as comparator
22	Titi MA, Alotair HA, Fayed A, et al. Effects of Computerised Clinical Decision Support on Adherence to VTE Prophylaxis Clinical Practice Guidelines among Hospitalised Patients. International Journal for Quality in Health Care 2021; 33: 22. DOI: https://dx.doi.org/10.1093/intqhc/mzab034.	No outcome of interest
23	Minary, A., Michel, B., Gourieux, B., & Vogel, T. (2020). Anticoagulant and antiplatelet combined therapy in patients 75 years and over with atrial fibrillation: a prospective observational study assessing adherence to clinical guidelines. European Journal of Hospital Pharmacy Science & Practice, 27(2), 84-89. doi:https://dx.doi.org/10.1136/ejhpharm-2018-001520	No outcome of interest
24	Jajosky, J., Howell, S. M., Honaker, J., Moriarty, A., & Shea, J. M. (2019). Improving Adherence to Practice Guidelines for Anticoagulation in Patients Receiving Neuraxial Anesthesia Using an Electronic Order Entry Alert System. Journal of patient safety, 15(3), 218-223. doi:https://dx.doi.org/10.1097/PTS.0000000000000219	No outcome of interest
25	Khalid, U., Bandeali, S., Jones, P. G., Virani, S. S., Hira, R., Hamzeh, I., . . . Alam, M. (2019). Prescription Patterns of Clopidogrel, Prasugrel, and Ticagrelor After Percutaneous Coronary Intervention With Stent Implantation (from the NCDR PINNACLE Registry). American Journal of Cardiology, 124(12), 1807-1812. doi:https://dx.doi.org/10.1016/j.amjcard.2019.09.003	No outcome of interest
26	Koolian, M., Kerzner, R., Kampouris, N., Mantzanis, H., & Susan, K. (2020). Antithrombotic stewardship: A novel approach to appropriate anticoagulant prescription. Research and Practice in Thrombosis and Haemostasis, 4(SUPPL 1), 1241-1242. doi:10.1002/rth2.12393	No outcome of interest
27	Krackhardt, F., Waliszewski, M., Kocka, V., Tousek, P., Janek, B., Hudec, M., . . . Pansieri, M. (2020). Real-World Dual Antiplatelet Therapy Following Polymer-Free Sirolimus-Eluting Stent Implantations to Treat Coronary Artery Disease. Cardiovascular Drugs & Therapy, 34(3), 335-344. doi:https://dx.doi.org/10.1007/s10557-020-06963-5	No outcome of interest
28	Krackhardt, M. F., Waliszewski, M., Pansieri, M., Lozano, F., Heang, T. M., Hudec, M., . . . Rischner, J. (2020). TCT CONNECT-249 Real-World Dual Antiplatelet Therapy Following Polymer-free, Sirolimus-Eluting Stent Implantations to Treat Coronary Artery Disease. Journal of the American College of Cardiology, 76(17), B110. doi:10.1016/j.jacc.2020.09.266	No outcome of interest

#	Full reference in alphabetical order (cut n paste from EndNote Preview)	Pre-coded reason
29	Barben, J., Menu, D., Rosay, C., Vovelle, J., Mihai, A. M., Nuss, V., . . . Manckoundia, P. (2020). The prescription of direct oral anticoagulants in the elderly: An observational study of 19 798 Ambulatory subjects. International Journal of Clinical Practice, 74(1), e13420. doi:https://dx.doi.org/10.1111/ijcp.13420	No outcome of interest
30	Murphy, A., Kirby, A., & Bradley, C. (2020). Monitoring of atrial fibrillation in primary care patients prescribed direct oral anticoagulants for stroke prevention. Irish Journal of Medical Science, 189(3), 961-966. doi:10.1007/s11845-019-02150-0	No outcome of interest
